# Decision regret in patients with rectal cancer undergoing multimodal therapy

**DOI:** 10.1016/j.ctro.2026.101211

**Published:** 2026-06-01

**Authors:** Tim Werfel, Alexander Rühle, Clemens Seidel, Julia Reuter, Andreas Hinz, Maximilian Römer, Nils H. Nicolay, Klaus Pietschmann, Aladdin Ali Deeb, Drilon Haziri, Georg Wurschi

**Affiliations:** a Department of Radiotherapy and Radiation Oncology, Jena University Hospital, Am Klinikum 1, 07747 Jena, Germany; b Comprehensive Cancer Center Central Germany (CCCG), Partner Site Jena, Am Klinikum 1, 07747 Jena, Germany; c Department of Radiation Oncology, University Medical Center Leipzig, Stephanstraße 9a, 04103 Leipzig, Germany; d Comprehensive Cancer Center Central Germany (CCCG), Partner Site Leipzig, Stephanstraße 9a, 04103 Leipzig, Germany; e Department of Medical Psychology and Medical Sociology, University Medical Center Leipzig, Philipp-Rosenthal-Straße 55, 04103 Leipzig, Germany

**Keywords:** Decision regret, Rectal cancer, Patient-reported outcomes, Multimodal therapy, Survivorship

## Abstract

**Purpose:**

This study examines decision regret (DR) in patients with locally advanced rectal cancer (UICC II/III) after multimodal treatment. We assessed prevalence, risk factors, and the impact of treatment schedules on DR.

**Methods:**

In this bicenter cross-sectional observational study, DR was measured using the 5-item Decision Regret Scale. Additional validated questionnaires assessed quality of life (QoL), fear of progression (FoP) and recurrence (FoR), depression and anxiety, social support, and perceived participation in decision-making (PDM). Parametric tests and linear regression were used for group comparisons and predictor analysis.

**Results:**

157 patients were included in the final analysis (mean age 68.8 ± 9.4 years; 67% male). The mean interval between radiotherapy (RT) and survey completion was 35.1 ± 19.8 months; 76.6% underwent surgical resection and 23.4% organ-preserving treatment. DR following RT was reported from 111 patients (70.7%), with 50 patients (31.8%) showing severe DR (>25 points). DR was correlated with FoP (r = 0.26, p < 0.001), FoR (r = 0.29, p < 0.001), and psychological distress (r = 0.28, p < 0.001). Conversely, DR was negatively correlated with PDM (r =  − 0.23, p = 0.004) and QoL (r =  − 0.40, p < 0.001). DR did not differ significantly between organ-preserving and trimodal treatment (p = 0.095) or between treatment modalities (p = 0.821). Multivariable regression analysis revealed lower global QoL (p = 0.026) and PDM (p = 0.019) to be independently associated with DR.

**Conclusions:**

DR was observed in a considerable proportion of patients in our cohort, underscoring its clinical relevance. DR was independently associated with worse QoL and lower PDM. Future studies should examine predictors of DR in larger, longitudinal cohorts and assess interventions to reduce DR.

## Highlights

• 31.8% of patients experienced strong decision regret.
• Lower quality of life and lower patient involvement were independently associated with higher regret.
• Organ preservation was not associated with higher regret.
• Decision regret did not differ between treatment modalities.

## Graphical abstract

**Figure:**
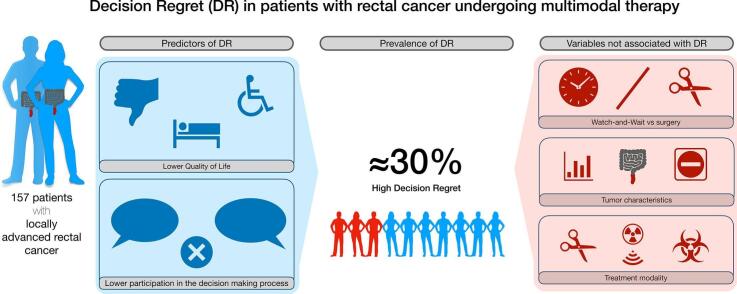

## Introduction

Decision regret (DR) is a negative cognitive-affective response following a decision-making process when outcomes fall short of expectations, and alternative choices are perceived as more favorable [1], [2]. This emotion may be triggered by situations involving suboptimal medical outcomes or decisions that are perceived as inappropriate [3], [4].

Not only does DR have a significant impact on patients' quality of life (QoL), but it can also substantially alter clinical outcomes [5]. As treatment decisions in oncology greatly impact patients' lives, dissatisfaction and DR are to be expected in some patients [6]. Effective communication and shared decision-making (SDM) are therefore essential [4], [7], [8], [9], [10]. DR has been documented across diverse oncology settings [5], [11], [12], [13], [14], [15], [16], [17].

Rühle et al. evaluated DR in patients with various tumor types treated with radiotherapy (RT). Nearly 40% experienced mild DR while about 20% reported severe DR. Therefore, more than half of the analyzed patients (56%) showed some degree of DR, highlighting its significance [18]. However, of the 207 patients, only 4.3% (n = 9) had rectal cancer, severely underrepresenting this group.

Rectal cancer is particularly notable in this context, as multiple therapeutic concepts might be suitable, each associated with distinct risk profiles [19], [20]. Beyond the primary treatment intent, additional critical considerations include the preservation or restoration of normal bowel function and anal continence, as well as the recovery of genitourinary function [21].

Locally advanced rectal cancer (UICC stage II/III) is routinely treated with multimodal therapies, including radiotherapy (RT), chemotherapy (CT) and resection. In high-risk cases (e.g., high-risk tumors, e.g., T4; localization in the lower third, involvement of the mesorectal fascia or extra-mesorectal vascular invasion), the treatment regimen can be intensified by adding neoadjuvant chemotherapy, which is referred to as a total neoadjuvant therapy (TNT) approach [20]. TNT is associated with improved tumor control and higher rates of complete response, enabling organ preservation in selected patients [22]. This results in a broad spectrum of available treatment strategies. Balancing oncological control and QoL can be highly challenging. Consequently, determining an individualized treatment strategy represents a complex clinical decision-making process, as no universally applicable treatment algorithm exists [20], [21]. Whether certain treatments contribute to DR, particularly when comparing patient subgroups managed with a ‘watch-and-wait’ strategy versus those undergoing standard surgical resection, remains to be systematically explored. This study investigates the prevalence of DR and associated potential risk factors, including different treatment sequences, in rectal cancer patients. Using validated patient-reported instruments in an observational, multicenter design, this work provides novel data in an underexplored area and may inform future research on patient-reported treatment experiences.

## Materials and methods

### Study design

This cross-sectional observational study was conducted between April 2025 and June 2025 at two tertiary radiotherapy facilities in central Germany using standardized questionnaires.

Inclusion criteria were (i) RT of rectal cancer with curative intent between 01/2019–12/2024, (ii) the ability to understand the German questionnaire, (iii) at least one clinical follow-up and (iv) informed consent.

Initially, potential study participants were invited to take part in the research through telephone contact. Paper-based questionnaires were used for data collection; informed consent was provided from all involved patients. Data on patient demographics and treatment details were retrospectively extracted from electronic medical records.

The study was approved by the local ethics committees of the Faculty of Medicine at University Hospital Leipzig (reference number: 077/23-ek) as well as the Faculty of Medicine at Jena University Hospital (reference number: 2025–3746-Bef). The study was conducted in accordance with the Declaration of Helsinki and is reported in accordance with the STROBE guidelines for cross-sectional studies.

### Endpoints and applied questionnaires

DR following RT served as the primary endpoint for this analysis and was assessed with the German version of the Decision Regret Scale (DRS) [18], [23], consisting of five sub-questions. Responses were recorded on a five-point Likert scale ranging from 1 = “I agree” to 5 = “I don’t agree,” with items 2 and 4 reverse scored. DR was assessed separately for RT, and, if applicable, for simultaneous CT and surgery. Following the original validation, the total DRS score was transformed to a scale ranging from 0 to 100. In accordance with previous DRS studies, a score of 0 indicated no DR, 1–25 indicated mild DR, and a score > 25 indicated strong DR [24]. However, no universally accepted cutoff for clinically relevant DR has been established, and the categorization should therefore be interpreted with caution. Secondary endpoints included DR following CT and surgery, QoL, Fear of Progression (FoP), social support and other psychological factors.

General QoL was measured using the EORTC QLQ-C30 questionnaire [25] and scored according to the official manual [26]. The validated QLQ-C30 summary score (C30-SS) was computed from 13 functioning and symptom scales (excluding global QoL and financial difficulties) when at least 10 scales were available and used for analyses [27].

FoP was accessed using the short form of the “Fear of Progression questionnaire” (FoP-12) [28]. Fear of Recurrence (FoR) was evaluated using the Concerns about Recurrence Questionnaire (CARQ-4) [29]. Depression and anxiety were captured with the Patient Health Questionnaire (PHQ-4) [30]. Social support was assessed using the ENRICHD Social Support Instrument (ESSI) [31]. Perceived participation in decision making (PDM) was measured with the Shared Decision-Making Questionnaire (SDM-Q-9) [32]. Furthermore, general demographic data were obtained via patient-reported questionnaires. Tumor risk groups were defined according to the 2017 ESMO tumor risk classification [33].

### Statistical analysis

Parametric descriptive analyses were used to characterize the study population. Questionnaire scores were transformed according to established scoring manuals to ensure comparability. Internal consistency was evaluated using Cronbach’s α. Subsequently, descriptive analyses and bivariate testing were conducted using Pearson’s correlation, independent samples t-tests, and one-way ANOVA. Subgroup analyses comparing treatment modalities were examined using repeated measures ANOVA. Although some variables showed moderate deviations from normality, parametric methods were considered appropriate following a consultation with an experienced biostatistician (H.A.), given their robustness in larger samples.

Significant predictors from the bivariate analyses were entered into a primary multivariable linear regression model (M0), together with the prespecified core covariates age and sex. Employment status was excluded from M0 due to operationalization-dependent significance. A sensitivity analysis was performed by adding employment status as a three-level categorical predictor (M1), followed by formal model comparison (ΔR^2^, AIC, BIC). Multicollinearity was evaluated using variance inflation factors (VIF), which indicated stable coefficients. Finally, residual diagnostics confirmed that assumptions of linear regression were met.

Missing values were handled according to standardized procedures. For score transformation, scale means were calculated only if at least 75% of items were completed. For bivariate analyses, pairwise deletion was applied to maximize sample size. In multivariable regression models, listwise deletion was used to ensure consistency across predictors.

A two-sided significance level of p < 0.05 was applied without correction for multiple testing, as all analyses were considered explorative and hypothesis-generating. Therefore, p values should be interpreted descriptively. Statistical analyses and visualization were conducted with JASP v0.19.3 (JASP Team, 2025), SPSS 29.0 (IBM SPSS Statistics, Armonk, NY / USA), and Numbers v14.4 (Apple Inc., Cupertino, CA / USA).

## Results

### Study cohort

**Fig. 1:**
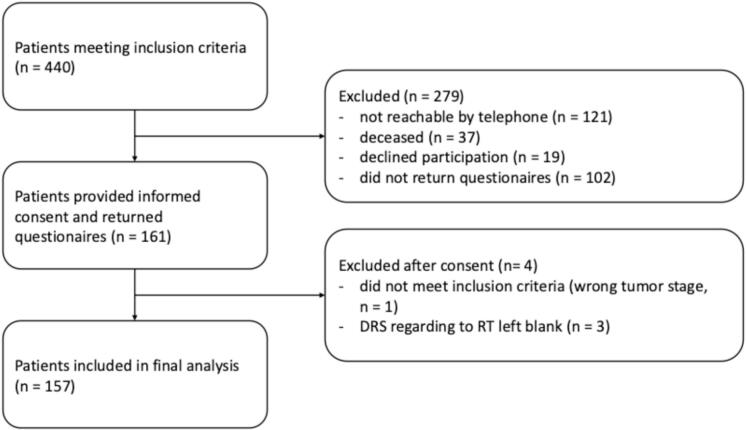
Study Flow Chart. Flow diagram illustrating patient selection, questionnaire distribution and final study population. Abbreviations: DRS = decision regret scale, RT = radiotherapy.

Of 440 eligible patients, 263 (60%) consented to receive paper-based questionnaires by mail, 161 returned complete questionnaires. The final study population included 157 patients with a mean age at diagnosis of 68.8 years (SD 9.43; range 30–88) and 105 (66.9%) patients being male. Reasons for exclusion are shown in Fig. 1.

The average time from first RT to questionnaire completion was 35.1 ± 19.8 months. 132 patients had UICC stage III and 25 had stage II disease. Tumors were predominantly located in the middle or lower rectum (n = 152). Most patients received long-course RT (n = 140) and concomitant CT (n = 135). Surgical resection was performed in 118 patients (76.6%), while 36 (23.4%) underwent organ preservation. Additional characteristics are summarized in Table 1.

**Table 1:** Demographic and clinical characteristics.

	*Valid*	*Missing*	*Mean (± SD)*	
**Age at study participation**	157	0	68.8 (± 9.4)	
**Age at first diagnosis**	157	0	65.7 (± 9.0)	
**Time between last fraction of RT and study participation**	157	0	35.1 (± 19.8)	
***Risk group***			***n***	***%***
very early			0	0,0
early			3	1,9
intermediate			40	25.5
advanced			74	47.1
bad			14	8.9
missing			26	16.6
***Sex***				
Male			105	66.9
Female			52	33.1
***marital status at study inclusion***				
single			15	9.6
married			106	67.5
divorced / separated			13	8.3
widowed			20	12.7
missing			3	1.9
***Living with a partner***				
yes			116	73.9
no			37	23.6
missing			4	2.5
***Highest school qualification***				
Lower secondary school leaving certificate (*Hauptschulabschluss*)			39	24.8
Intermediate secondary school certificate (*Realschulabschluss*)			62	39.5
Entrance qualification for university of applied sciences (*Fachhochschulreife*)			14	8.9
General university entrance qualification (*Abitur*)			33	21.0
Other			7	4.5
missing			2	1.3
***Having children under the age of 14***				
yes			4	2.5
no			152	96.8
missing			1	0.6
***Employment status at time of study participation***				
employed			32	20.4
unemployed			3	1.9
Retired			120	76.4
missing			2	1.3
***Tumor stage (UICC) at time of RT***				
UICC II			25	15.9
UICC III			132	84.1
***Concomitant CT***				
yes			135	86.0
no			22	14.0
***Primary tumor location (rectum)***				
upper third			5	3.2
middle third			84	53.5
lower third			68	43.3
***Tumor resection***				
yes			118	75.2
no			36	22.9
missing			3	1.9
***Tumor recurrence***				
yes			16	10.2
no			139	88.5
missing			2	1.3
***Treatment setting of RT***				
neoadjuvant			143	91.1
adjuvant			14	8.9
***RT regimen***				
Short Course			17	10.8
Long Course			140	89.2

*Values are presented as mean ± SD or n (%), as appropriate. Abbreviations: RT = radiotherapy; CT = chemotherapy*.

### Prevalence and associations with DR

DRS was assessed separately for RT, CT, and surgery. For RT, 46 patients (29.3%) reported no DR, 61 (38.9%) mild DR, and 50 (31.8%) strong DR.

**Fig. 2:**
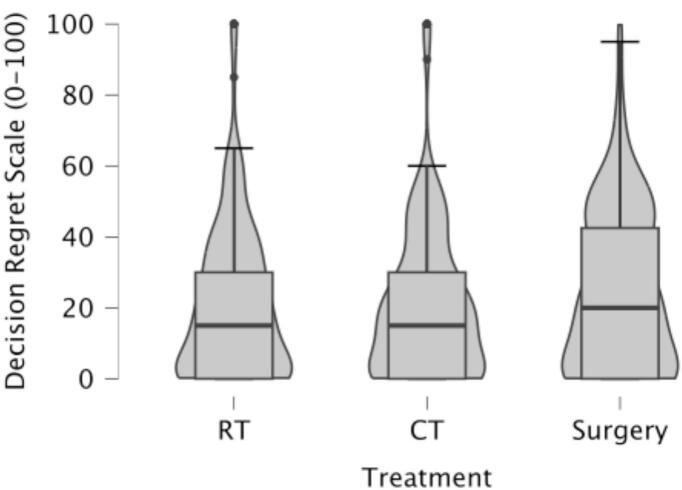
Decision Regret across treatment modalities. Distribution of decision regret scores across treatment modalities. Violin plots show the distribution of decision regret scores; boxes represent median, interquartile range. Abbreviations: RT = radiotherapy, CT = chemotherapy.

Mean DRS was 20.4 ± 21.6 for RT, 20.0 ± 21.1 for CT, and 23.7 ± 22.5 for surgery. Repeated measures ANOVA revealed no statistically significant differences of the DRS between the three treatment modalities (repeated-measures ANOVA, Greenhouse Geisser corrected, p = 0.821). Pairwise comparisons were all non-significant. Analyses were based on 72 cases, as listwise deletion was applied for missing values. Fig. 2 displays DR across treatment modalities.

Pearson’s correlations demonstrated significant positive associations of DR regarding RT with FoP (r = 0.26, p = 0.001), FoR (r = 0.29, p < 0.001), and depression as well as anxiety (PHQ-4; r = 0.28, p < 0.001). Financial difficulties (r = 0.31, p < 0.001) were also linked to increased DR. In contrast, DR showed a negative correlation with PDM (r =  − 0.23, p = 0.004) and C30-SS (r =  − 0.40, p < 0.001).

In further bivariate analyses using t-tests and ANOVA, no significant associations were observed for tumor recurrence, sex, or UICC stage (all p > 0.05). DR did not differ significantly between organ-preserving and trimodal treatment (t(152) =  − 1.68, p = 0.095). Complete results of bivariate testing are provided in Tables 2*,* S1 and S2.

**Table 2:** Results of independent samples t-tests comparing decision regret across selected patient and treatment characteristics.

	Comparison	t	df	p	Coheńs d	95% CI
Resection	yes vs no	−1.68	152	0.095	−0.32	−0.69 – 0.06
Tumor Recurrence	yes vs no	0.61	153	0.543	0.16	−0.36 – 0.68
Concomitant chemotherapy	yes vs no	1.34	155	0.182	0.31	−0.14 – 0.76
Sex	Male vs female	−0.54	155	0.593	−0.09	−0.42 – 0.24
UICC	II vs III	0.35	155	0.728	0.08	−0.35 – 0.50
Living with a steady partner	yes vs no	0.08	151	0.934	0.02	−0.35 – 0.39
RT regimen	Short vs long course	0.57	155	0.571	0.15	−0.36 – 0.65

*Independent samples t-tests were used. Cohen’s d is reported as effect size. Abbreviations: RT = radiotherapy*.

In the prespecified multivariable model, only C30-SS (p = 0.026, β = -0.27) and PDM (p = 0.019, β = -0.19) were independently associated with DR. Other variables, including FoP, FoR and PHQ, showed no significant associations (Table 3). In an additional multivariable model (M1), including employment status as sensitivity analysis, C30-SS (p = 0.045) and PDM (p = 0.017) remained significantly associated (Supplementary Table S3)*.* As the model fit of M1 was not significantly improved over M0 (ΔR^2^ = 0.019, p = 0.172; ΔAIC = 0.15; BIC increased; Supplementary Table S4), the more parsimonious model (M0) was retained to limit overfitting and increase model stability.

**Table 3:** Multivariable linear regression model (M0) examining associations between clinical and patient-reported variables and DR.

	Standardized Beta	p
Fear of progression	0.02	0.894
Fear of recurrence	0.12	0.291
Anxiety	0.04	0.696
Depression	−0.06	0.611
Participatory decision making (PDM)	−0.19	0.019
QoL Summary Score (C30-SS)	−0.27	0.026
Social Support	−0.07	0.383
FI	0.14	0.118
Age at diagnosis	0.11	0.193
Sex	0.04	0.583
	
final model: R^2^ = 0.26		

Linear regression analysis, model 0 (M0) represents the baseline model.

## Discussion

DR has been reported as a relevant issue among patients with cancer [18]. Importantly, DR has been added as a secondary endpoint in ongoing trials such as STARTREC-3 [34], indicating the growing recognition as a patient-reported outcome measure (PROM) in rectal cancer care. However, its role in patients with locally advanced rectal cancer undergoing multimodal therapy remains insufficiently explored. To our knowledge, this study represents the first systematic examination of DR in this patient population. DR was observed in a substantial proportion of our cohort, with approximately two-thirds reporting some degree of DR and 32% experiencing high levels. Higher DR was consistently associated with poorer QoL and reduced PDM, whereas clinical variables such as tumor stage or resection status were not significant predictors. The linear regression model showed only modest explanatory power. Given the multifactorial nature of PROMs, a substantial proportion of variance remained unexplained. Furthermore, the exploratory regression model was primarily intended to adjust for potential confounders within this non-randomized cohort and should therefore be considered hypothesis-generating rather than predictive. Nevertheless, the identified associations may still contribute to a better understanding of the investigated outcome.

In our analysis, DR did not differ significantly between the treatment modalities RT, CT, or surgery, which is consistent with the findings of Tews et al. in head and neck cancer patients [17]. It may be difficult for patients to attribute later symptoms or side effects to specific treatment components, which may reduce the likelihood of modality-specific DR. Furthermore, Meyer et al. found no significant difference in DR between rectal cancer patients undergoing delayed versus immediate resection, although their study cohort was relatively small (n = 51) [35]. This suggests that the type of treatment itself may be less relevant for the development of DR than patient-centered factors such as health-related QoL or satisfaction with the SDM. It therefore appears plausible that patients retrospectively evaluate not the medical procedure itself but rather its long-term impact on daily functioning and the quality of interaction with healthcare professionals.

In line with this, C30-SS proved to be significantly associated with DR in the multivariable regression model, similar to previous studies on DR in cancer patients [13], [17], [36], [37]. Patients with lower reported QoL often experience an increased symptom burden [38]. This may contribute to a more negative retrospective evaluation of treatment decisions. Reduced QoL is frequently accompanied by diminished social interaction and isolation [39], [40], fostering intrusive thought patterns that may further facilitate DR [41]. Lower QoL may also reduce resilience and coping capacity [42], thereby increasing vulnerability to negative evaluations of past decisions [43]. These mechanisms may help explain the observed association of C30-SS with DR. However, no causal relationship can be inferred from our data. While impaired QoL may predispose patients to stronger DR, DR itself may also negatively affect perceived QoL and treatment satisfaction. In addition, conceptual overlap between these PROMs may have contributed to the observed association.

Nevertheless, routine assessment of QoL and DR using standardized instruments may improve understanding of PROMs and help identify patients who may benefit from supportive interventions. Early and continuous QoL assessment (e.g. EORTC QLQ-C30), systematic screening of DR using the DRS and targeted interventions to improve QoL [44] may additionally improve patient experience. Further studies are needed to confirm the association between improved QoL and lower DR.

Besides QoL, perceived PDM showed an independent association with DR in our multivariable analysis. This finding is in line with previous studies reporting lower levels of DR in patients who felt actively involved in the decision process [3], [12], [37], [45], [46], [47]. These findings suggest that patients reporting lower PDM also tended to report higher DR. Integration into SDM enhances patients’ sense of autonomy and responsibility [48], which in turn may reduce retrospective DR. DR might arise less from the treatment itself and more from a discrepancy between expectations and actual outcomes [46]. Greater involvement in SDM helps establish more realistic expectations by improving patients’ understanding of their condition, treatment options, and potential complications [49]. Consequently, higher SDM may be linked to DR by aligning expectations with clinical reality. In contrast, some studies have reported higher DR among patients who perceived a greater share of responsibility in the decision-making process [50], [51]. This suggests that the impact of PDM may be moderated by factors such as health literacy or decisional preparedness. Joshua et. al. concluded, that in their cohort of rectal cancer patients, a different role in the SDM as preferred by the patient increased the likelihood of DR [52]. Therefore, assessment of health literacy and tailoring of the decision-making process to individual needs should be considered in clinical practice [53], [54]. Several strategies have proven effective in strengthening SDM, including the use of evidence-based decision aids [49], [55] and communication training for healthcare professionals [56]. Our findings suggest that these interventions could be helpful in reducing DR in rectal cancer patients.

No significant difference in DR was observed between patients managed with a watch-and-wait strategy after TNT and those undergoing standard surgical resection. This is consistent with our overall finding that DR did not differ between treatment modalities (RT, CT, or surgery). In our cohort, DR appeared to be less driven by the type of therapy but by other factors. In contrast, Windon et al. reported that in their cohort of head and neck cancer patients, DR increased by 13 points with each additional treatment modality. Therefore, patients experienced higher DR after surgery with adjuvant RT compared to surgery or RT alone [57]. Based on our cohort, it appears plausible that SDM may lessen modality-specific differences in DR, as patients might evaluate their experiences more on subjective impressions than on objective clinical consequences. Furthermore, the watch-and-wait approach may be perceived as more daunting or uncertain [58], potentially neutralizing its expected benefit in terms of DR reduction. Prior psychological research highlights choice justification, whereby individuals retrospectively reframe decisions in a more positive light to reduce negative emotions as DR [59]. Although not yet examined in cancer care, such mechanisms may similarly influence the development of DR in this patient group. Consistent with this view, several studies suggest that subjective factors such as QoL, expectations, and perceived involvement often outweigh objective clinical variables [60], including treatment modality, possibly reflecting underlying psychological rationalization. It should be noted, however, that only 36 of 154 patients received organ-preserving treatment, resulting in an imbalanced group distribution.

These findings have important clinical implications. In rectal cancer, organ preservation may be achievable after TNT and should therefore be discussed transparently within SDM. However, if a complete response is not achieved, surgical resection remains necessary, which may lead to unmet expectations and increased DR. Managing expectations through clear, educative communication is therefore a key responsibility of healthcare professionals and may represent an important target for reducing DR.

The operationalization-dependent association of employment status indicates limited robustness. Despite statistical significance in one specification, its inclusion did not significantly improve overall model fit. These findings warrant cautious interpretation and require confirmation in larger prospective studies.

Several variables, including depression, anxiety, FoP and financial difficulties were associated with DR in bivariate analyses but not after adjustment, suggesting overlapping influences. Given their established relevance in other tumor entities [17], [61], [62], these factors may still contribute to patients’ experience of DR and merit further investigation as potential moderators.

We acknowledge several limitations. Importantly, as a cross-sectional observational study, causal relationships between DR and the examined variables cannot be established. The herein presented results should be considered hypothesis-generating. The cross-sectional design prevents evaluation of temporal changes or the influence of comorbidities. Although the interval between RT and survey completion was not associated with DR, individual trajectories were not captured. Previous studies in other cancer types have reported both stable and increasing levels of DR over time [14], [63]. Second, the overall sample size of 157 patients, and particularly the relatively small number of subjects within the subgroups (e.g. organ preservation vs. surgery), limit the power and warrant cautious interpretation. The absence of significant differences between treatment modalities may partly reflect the limited statistical power in subgroup analyses. Furthermore, it remains unclear to what extent patients are able to distinguish and attribute DR to specific treatment components such as RT, CT or surgery. Third, as only German-speaking rectal cancer patients were included, comparability across countries and tumor entities may be restricted. Furthermore, recall bias cannot be excluded, given that the mean time between treatment and survey was 35.1 months. Prospective longitudinal studies may help reduce recall bias and provide a more detailed understanding of the development of DR over time. Given the moderate response rate, selection and non-response bias cannot be excluded, as patients with particularly strong positive or negative experiences may have been more likely to participate and no formal comparison between responders and non-responders was performed. As no adjustment for multiple testing was performed, the risk of type I error cannot be excluded. Additionally, the applied missing data strategy may have introduced bias if data were not missing completely at random. Finally, although we assessed a range of clinical and psychological variables, important factors such as health literacy, satisfaction with care and functional outcomes were not examined. As these factors have been shown to be relevant in previous research [12], [18], [53], [64], residual confounding cannot be excluded. To maintain a feasible questionnaire length, reduce participant burden and improve response rates, the number of assessed variables had to be limited.

## Conclusion

DR was observed in a substantial proportion of rectal cancer patients after multimodal treatment, with nearly one third reporting strong DR. DR was independently associated with QoL and PDM. No significant difference in DR was observed between patients undergoing organ preservation and those receiving traditional trimodal therapy.

## Declaration of generative AI and AI-assisted technologies in the manuscript preparation process

During the preparation of this work the author used ChatGPT (Open AI) to assist with language editing and manuscript preparation. After using this tool/service, the author reviewed and edited the content as needed and takes full responsibility for the content of the published article.

## Data availability statement

The data underlying this study are not publicly available due to patient privacy and institutional data protection regulations but are available from the corresponding author on reasonable request.

## CRediT authorship contribution statement

**Tim Werfel:** Writing – original draft, Visualization, Investigation, Formal analysis, Data curation, Conceptualization. **Alexander Rühle:** Writing – review & editing, Methodology, Conceptualization. **Clemens Seidel:** Writing – review & editing, Methodology, Conceptualization. **Julia Reuter:** Writing – review & editing, Investigation, Data curation. **Andreas Hinz:** Writing – review & editing, Methodology, Formal analysis. **Maximilian Römer:** Writing – review & editing, Investigation. **Nils H. Nicolay:** Writing – review & editing, Resources. **Klaus Pietschmann:** Writing – review & editing, Resources. **Aladdin Ali Deeb:** Writing – review & editing, Investigation. **Drilon Haziri:** Writing – review & editing, Investigation. **Georg Wurschi:** Writing – review & editing, Writing – original draft, Supervision, Methodology, Conceptualization.

## Funding

This research did not receive any specific grant from funding agencies in the public, commercial, or not-for-profit sectors.

## Declaration of competing interest

The authors declare that they have no known competing financial interests or personal relationships that could have appeared to influence the work reported in this paper.

## Appendix A Supplementary data

Supplementary Tables S1-S4 Additional statistical analyses including Pearson correlation analyses, one-way ANOVA, multivariable regression results, and regression model comparison.
