# Supplementary material for: Decision regret in patients with rectal cancer undergoing multimodal therapy
Source: Clin Transl Radiat Oncol. 2026 Jun 1;59:101211. doi: 10.1016/j.ctro.2026.101211 (PMC13251190; doi:10.1016/j.ctro.2026.101211)
Supplement: Supplementary Tables S1-S4 — Additional statistical analyses including Pearson correlation analyses, one-way ANOVA, multivariable regression results, and regression model comparison. [file mmc1.docx]

**Table S1: Pearson correlation matrix showing associations between DR and patient-reported outcome measures.**

|  | **Pearson's r** | **p** | **95% CI** |
| --- | --- | --- | --- |
| Anxiety | 0.25 | 0.001 | 0.10 – 0.39 |
| Depression | 0.26 | < 0.001 | 0.11 – 0.41 |
| PHQ4 total | 0.28 | < 0.001 | 0.13 – 0.42 |
| Fear of Progression | 0.26 | 0.001 | 0.11 – 0.40 |
| Fear of Recurrence | 0.29 | < 0.001 | 0.14 – 0.42 |
| Social Support | -0.19 | 0.015 | -0.34 – -0.04 |
| Participatory decision making (PDM) | -0.23 | 0.004 | -0.37 – -0.08 |
| Age at first diagnosis | 0.11 | 0.191 | -0.05 – 0.26 |
| QoL Summery Score (C30-SS) | -0.40 | < 0.001 | -0.53 – -0.26 |
| Financial burden | 0.31 | < 0.001 | 0.16 – 0.44 |
| Time between RT and questionnaire | 0.03 | 0.699 | -0.13 – 0.19 |

*Pearson correlation coefficients (r) are reported. Abbreviations: DR = decision regret; RT = radiotherapy*

**Table S2: One-way ANOVA comparing DR following RT**

|  | **Comparison** | **F (df_between, df_within)** | **p** | η² |
| --- | --- | --- | --- | --- |
| Tumor location | Lower, middle and upper third | F(2, 154) = 2.76 | 0.066 | 0.03 |
| Marital status | categorical | F(3, 150) = 0.07 | 0.978 | <0.01 |
| Educational attainment | categorical | F(4, 150) = 1.45 | 0.219 | 0.04 |
| Employment status | Employed, unemployed, retired | F(2, 152) = 3.94 | 0.021 | 0.05 |

*One-way ANOVA was used. Effect sizes are reported as η². Abbreviations: DR = decision regret; RT = radiotherapy*

**Table S3: linear regression analysis of DR following RT including employment status (M1)**

|  | **Standardized Beta** | **p** |
| --- | --- | --- |
| Fear of progression | 0.04 | 0.752 |
| Fear of recurrence | 0.11 | 0.349 |
| Anxiety | 0.02 | 0.876 |
| Depression | -0.05 | 0.720 |
| Participatory decision making (PDM) | -0.19 | 0.017 |
| QoL Summery Score (C30-SS) | -0.24 | 0.045 |
| Social Support | -0.07 | 0.428 |
| FI | 0.14 | 0.135 |
| Age at diagnosis | -0.06 | 0.621 |
| Sex | 0.01 | 0.955 |
| Employment status | 0.18 | 0.063 |
|  | |  |
| final model: R² = 0.28 | |  |

*Employment status showed an operationalization-dependent association with DR in bivariate analyses but was not significant in the multivariable model. Abbreviations: DR = decision regret; RT = radiotherapy*

**Table S4: Model comparison between the primary regression model (M0) and the model including employment status (M1).**

| **Model** | **N** | **R²** | **Adjusted R²** | **AIC** | **BIC** |
| --- | --- | --- | --- | --- | --- |
| M0 | 152 | 0.263 | 0.211 | 1334.21 | 1370.42 |
| M1 | 151 | 0.282 | 0.219 | 1334.36 | 1376.60 |

*Model comparison showed that inclusion of employment status did not significantly improve model fit (ΔR² = 0.019, p = 0.172), with both AIC (ΔAIC = 0.15) and BIC increasing.*
